# Mitochondrial dysfunction in Parkinson disease: evidence in mutant *PARK2* fibroblasts

**DOI:** 10.3389/fgene.2015.00078

**Published:** 2015-03-11

**Authors:** Maria C. Zanellati, Valentina Monti, Chiara Barzaghi, Chiara Reale, Nardo Nardocci, Alberto Albanese, Enza M. Valente, Daniele Ghezzi, Barbara Garavaglia

**Affiliations:** ^1^Unit of Molecular Neurogenetics – Pierfranco and Luisa Mariani Center for the Study of Mitochondrial Disorders in Children, Foundation of the Carlo Besta Neurological InstituteIRCCS, Milan, Italy; ^2^Unit of Child Neurology, Foundation of the Carlo Besta Neurological InstituteIRCCS, Milan, Italy; ^3^Neurology Unit, Foundation of the Carlo Besta Neurological InstituteIRCCS, Milan, Italy; ^4^Casa Sollievo Della Sofferenza Hospital, Mendel InstituteRome, Italy

**Keywords:** parkin, *PARK2*, mitochondrial membrane potential, oxygen consumption, mitochondrial dynamics

## Abstract

Mutations in *PARK2*, encoding Parkin, cause an autosomal recessive form of juvenile Parkinson Disease (JPD). The aim of the present study was to investigate the impact of *PARK2* mutations on mitochondrial function and morphology in human skin fibroblasts. We analyzed cells obtained from four patients clinically characterized by JPD, harboring recessive mutations in *PARK2.* By quantitative PCR we found a reduction (<50%) of *PARK2* transcript in all patients but one; however Western Blot analysis demonstrated the virtual absence of Parkin protein in all mutant fibroblasts. Respiration assays showed an increment of oxygen consumption, which was uncoupled to ATP cellular levels. This finding was probably due to presence of altered mitochondrial membrane potential (ΔΨ_m_), confirmed by JC-1 analysis. The mitochondrial network was comparable between mutant and control cells but, interestingly, a “chain-like” network was found only in mutant fibroblasts. Dissipation of ΔΨ_m_ usually leads to mitochondrial fragmentation in healthy cells and eventually to mitophagy; however, this behavior was not observed in patients' fibroblasts. The absence of mitochondrial fragmentation in mutant Parkin fibroblasts could results in accumulation of damaged mitochondria not targeted to mitophagy. This condition should increase the oxidative stress and lead to cellular dysfunction and death. Our results suggest that *PARK2* mutations cause mitochondrial impairment, in particular reduction in ATP cellular levels and alteration of ΔΨ_m_, even in non-neuronal cells and confirm the hypothesis that Parkin holds a pivotal role in pro-fission events.

## Introduction

Parkinson's disease (PD) is the second most common neurodegenerative disorder. Although its etiology is mainly elusive, it has been shown that some substances with toxic effect on mitochondrial functioning (e.g., MPTP and rotenone) can induce parkinsonism in human and animal model (Langston et al., 1983; Nicklas et al., 1985), suggesting an important role of mitochondria in the pathophysiology of PD. Nevertheless, even if environment factors play an important role in PD, mutations in a number of genes have been found to cause inherited forms of PD, with both autosomal dominant (e.g., *LRRK2, SNCA)* and recessive transmission (e.g., *PARK2, PINK1, DJ1*). Many of these PD gene-products have also been proven to influence mitochondrial bioenergetics and dynamics, including membrane potential, respiratory activity, cristae structure, calcium homeostasis, mitochondrial DNA (mtDNA) integrity and clearance of dysfunctional mitochondria (reviewed in Sai et al., 2012).

Mutations in *PARK2*, encoding Parkin, are the most frequent cause of juvenile PD (JPD), accounting for up to 50% of the cases with an age of onset <40 years (Kitada et al., 1998).*PARK2* mutations range from single base pair substitutions, splice site mutations and small nucleotide deletions, to large deletions or duplications of one or more *PARK2* exons; albeit through different mechanisms, all these variants probably have a “loss of function” effect.

Parkin is a multifunctional E3 Ubiquitin ligase, which is able to perform a variety of ubiquitin linkages associated with numerous cellular functions. To date, more than 30 putative substrates have been reported and Parkin regulates their activity through both degradative and non degradative ubiquitination (reviewed in Dawson and Dawson, 2010). Several studies have highlighted for Parkin a pivotal role in mitochondrial homeostasis and dynamics. In association with PINK1, Parkin acts in mitochondrial fission and fusion, mitochondrial transport and removal of damaged mitochondria through mitophagy process (Narendra et al., 2008; Wang et al., 2011; Yu et al., 2011; Ashrafi et al., 2014; Cook et al., 2014). The triggering mechanism of PINK1/Parkin-dependent mitophagy is the loss of mitochondrial transmembrane potential (ΔΨ_m_). A remodeling mechanism of mitochondrial network aiming at isolating damaged organelles from remaining healthy mitochondria and detaching them from cytoskeletal elements (Twig et al., 2008; Chan et al., 2011; Wang et al., 2011; Frank et al., 2012) is necessary to ensure that only damaged mitochondria are removed, and precedes mitophagy. A recent study of Buhlman et al. (2014) has demonstrated how Parkin may promote mitochondrial division by a mechanism that is dependent from Drp1, a GTPase that regulates mitochondrial fission, but seems independent from PINK1 activity and probably, also, not a prerogative for mitochondrial clearance. These findings collectively demonstrate that Parkin is intimately involved in preventing mitochondrial dysfunction.

Till now the studies performed on *PARK2* mutant fibroblasts in order to explore the impact of Parkin on mitochondrial functionality, have remain elusive (Mortiboys et al., 2008; Grünewald et al., 2010; Pacelli et al., 2011; van der Merwe et al., 2014); however these reports highlighted how fibroblasts derived from PD patients may be a reliable model system to study mitochondrial dysfunction. We report here that Parkin-mutant fibroblasts derived from PD patients showed alterations in mitochondrial bioenergetics, in particular reduction in ATP cellular levels, decrease of ΔΨm and probably impairment in mitochondrial fission. These data suggest that *PARK2* mutations cause mitochondrial dysfunction even in non-neuronal cells confirming that skin fibroblasts from *PARK2* mutant patients may be a suitable system to gain further details on cellular dysfunction underlying PD and possibly to test new therapeutic approaches.

## Materials and methods

The study was approved by the ethics committee of the Fondazione IRCCS (Istituto di Ricovero e Cura a Carattere Scientifico) Istituto Neurologico “Carlo Besta” and all individuals gave written, informed consent.

### Genetic studies

Genotyping was performed by direct DNA sequencing of all *PARK2* exons and intron-exon boundaries, and using the MLPA dosage kits (salsa MLPA kit P051-B1and P052-C1, MRC Holland) covering all exons of *PARK2*. The protocol used was per manufacturer's instructions. Phenotypic and genotypic data of PD patients and controls are summarized in Table 1.

**Table 1:** **Genotypic and phenotypic characterization of investigated individuals**.

	**Code**	**Sex**	**Age of onset (year)**	**Mutation**		**Clinical status**
				**DNA**	**Protein**	
Mutants	Pt1	F	35	c.714C>G c.823C>T	p.C238W p.R275W	Affected
	Pt2	M	41	c.101_102delAG c.101_102delAG	p.Q34R^*^5 p.Q34R^*^5	Affected
	Pt3	F	37	Deletion ex1 c.823C>T	No transcript/protein p.R275W	Affected
	Pt4	F	14	Duplication ex2 Deletion ex3-4-5	No transcript/protein p.N58_A206del	Affected
Controls	Ct1	F	/	None		Unaffected
	Ct2	F	/	None		Unaffected
	Ct3	M	/	None		Unaffected
	Ct4	F	/	None		Unaffected

*Refseqs used: NM_004562.2 (for DNA), NP_004553.2 (for protein). Exon (ex) deletion and duplication at DNA level were detected by MLPA dosage kits*.

### RNA extraction, PCR, and real time PCR analysis

Total mRNA was extracted from fibroblasts (80% confluence) using the RNeasy Mini Kit (Qiagen) according to the manufacture's protocol. RNA quantity was measured with the Nanodrop instruments (Nanodrop Technologies) and RNA integrity was verified through gel electrophoresis. One microgram of RNA was reverse-transcribed into cDNA by GoScript Reverse Transcriptase protocol (Promega). *PARK2* transcript was amplified using PCR with specific primers on *PARK2* 5′ and 3′ untranslated regions (UTRs). Primers sequences are as follows: PARK2 Fw 5′-GAGAGCCGCTGGTGGGAG-3′; Rc 5′-AAGTCCAACTACAGCCAAATTG-3′.

*PARK2* expression in cDNA samples was determined using quantitative PCR with specific amplicons and SYBR-green chemistry (GoTaq qPCR Master Mix, Promega); glyceraldehyde-3-phosphate dehydrogenase (*GAPDH*) was used for normalization. Primer sequences are as follows:

PARK2 (nucleotides 1138-1212) Fw 5′-CTGCGACACCACCACAG-3′; Rc 5′-TGGATTGCACTTGAATCTGTG-3′
PARK2 (nucleotides 1246-1338), Fw 5′-TGACCAGAGGAAAGTCACCTG-3′; Rc 5′-TCTTTACATTCCCGGCAGAA-3′; GAPDH, Fw 5′-CTCTGCTCCTCCTGTTCGAC-3′; Rc 5′-ACGACCAAATCCGTTGA-3′.

### Skin fibroblasts and culture condition

Skin biopsies were taken from patients and four healthy controls following standard clinical procedure. Skin fibroblasts were grown at 37°C under 5% CO_2_ humidified atmosphere in F14 (EuroClone) medium supplemented with 10% FBS (EuroClone), 4% L-Glutamine and 2% penicillin/streptomycin, 1.5% Glucose, 1% Insulin, 0.1% FGF, 0.1% EGF. Five days before the experiment the growth medium was changed to EMEM (EuroClone) supplemented with 10% FBS (EuroClone), 4% L-Glutamine and 2% penicillin/streptomycin. The cells used in present studies were between passages 7 and 12.

### Measurement of oxygen consumption and extracellular acidification rates

Measurements of endogenous respiration rates in intact cell were performed through SeaHorse XF96 Analyzer (Seahorse Bioscience). Cells are seeded at 20,000 cells/well in a 96-well SeaHorse culture plate and leave to adhere overnight. Growth medium was replaced with non-buffered DMEM and the plate was incubated at 37°C without CO_2_ for 30 min before starting the assay. Oxygen consumption (OCR) was measured under basal condition (OCR-B), after injection of oligomycin, an inhibitor of complex V (OCR-O), and after addition of the uncoupling agent FCCP, carbonyl cyanide 4-(trifluoromethoxy) phenylhydrazone (OCR-F). A representative trace of the OCR measurements was reported in Supplementary Figure S1. The readout of cellular respiration in different conditions can be used to define bioenergetic parameters, such as the maximal respiration rate (MRR) (Invernizzi et al., 2012). MRR, index of electronic chain transport efficiency, corresponds to OCR-F minus OCR-B.

The instrument also measures the extracellular acidification rate (ECAR).

The detailed protocol was as follows:

**Table:** 

Calibration/equilibration, 2 cycle:	1′ mixing
	3′ waiting
Basal, 4 cycle:	5′ mixing
	4′ recording (OCR-B and ECAR)
Oligomycin injection, 4 cycle:	5′ mixing
	4′ recording (OCR-O)
FCCP injection, 5 cycle:	5′ mixing
	4′ recording (OCR-F)

All values were normalized by means of cell counts performed through CyQuant Direct cell proliferation Assay kit (Invitrogen).

### ATP measurements

ATP levels were determined using the ATPlite Assay kit (PerkinElmer). This method is based on mono-oxygenation of luciferin, catalyzed by luciferase in the presence of Mg^2+^, ATP, and oxygen, resulting in a luminescent signal that is proportional to the ATP.

Fibroblasts were seeded in 96-well culture plates at count of 20,000 cells per individual. Luminescence was measured using a multilabel plate reader (PerkinElmer). Values were normalized by means of cell counts performed through CyQuant Direct cell proliferation Assay kit (Invitrogen) according to the manufacturer's protocol.

### Mitochondrial network and membrane potential analysis

Twenty thousand cells were seeded in 35 mm dish (Microtech) (70% confluence) and leave to adhere overnight. Mitochondrial morphology was assessed after cell staining with 10 nM Mitotracker CMX-Red (Invitrogen) for 30 min at 37°C. Fluorescence was visualized with a digital imaging system using an inverted microscope (Nikon, Japan). Images were captured with a Photometrics Cascade CCD camera system (Crisel) and analyzed with Metamorph acquisition/analysis software. The network morphology was assessed by Metamorph analysis: cells with shape factor ≥0.650 were classified as fragmented network, cells with shape factor <0.650 as tubular network.

Detection of altered mitochondrial membrane potential was performed using 5,5′,6,6′-tetrachloro-1,1′,3,3′ tetraethylbenzimidazolylcarbocyanine iodide (JC-1) staining kit (Sigma, CS0390) according to manufacturer's instruction.

### Western blot analysis

Approximately 1 × 10^6^ fibroblasts were trypsinized, centrifuged at 1200 rpm for 5 min, and solubilized in 200 μl of RIPA buffer (50 mM Tris-HCl [pH 7.5], 150 mM NaCl, 1% NP40, 0.5% NaDOC, 5 mM EDTA) with 1 × Complete Mini Protease Inhibitor Cocktail Tablets (Roche) for 40 min at 4°C. Thirty micrograms of proteins were used for each sample in denaturing sodium-dodecyl sulfate polyacrylamide gel electrophoresis (SDS-PAGE). Immunoblot analysis was performed with the ECL-chemiluminescence kit (Amersham) according to the manufacturer's protocol. A rabbit polyclonal anti-Parkin antibody was used at 1:700 dilution (Cell Signaling); a mouse monoclonal anti-Vinculin was used at 1:1000 dilution (Sigma). Secondary anti-rabbit and anti-mouse were used at 1:5000 and 1:3000, respectively.

### Statistical analysis

For individual experiments, data obtained were calculated as the mean of the replicates ± standard deviation (SD) and then data were compared using One-Way ANOVA followed by Tukey test (vassarstat.net). Although the measurements obtained from the replicates for each sample were relatively consistent in individual experiments, the values may vary in different experiments performed in different days. For this reason, we transformed each value of OCR, ECAR, OCR/ECAR ratio, MRR, and ATP concentration, into standard (z) scores, in order to make the data of three different experiments comparable with each other and perform statistical analysis to the entire collection of experimental values (Invernizzi et al., 2012).

For evaluation of mitochondrial potential and network analysis Chi^2^-Pearson test was used to compare controls and mutants.

## Results

### Fibroblasts derived from patients with mutations in *PARK2* have reduced level of *PARK2* mRNA and parkin protein compared to controls

**Figure 1:**
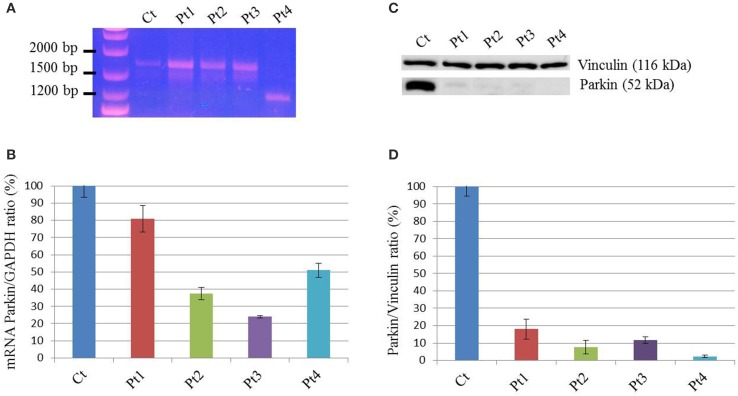
**mRNA and protein levels of Parkin. (A)** PCR amplification on cDNA obtained from mRNA extracted from patients (Pt) and control (Ct) fibroblasts, using primers in the 5′ and 3′-UTRs *PARK2* regions. The band corresponding to full-length transcript was present in Ct, Pt1, Pt2, and Pt3. A shorter transcript was amplified from Pt4, and sequence analysis showed that it corresponds to a *PARK2* isoform lacking exons 3-4-5. **(B)** Quantitative PCR analysis of *PARK2* transcript in controls (Ct) and mutant fibroblasts (Pt) normalized to GAPDH. The controls' mean is set equal to 100%. Data were from two independent experiments performed in duplicate. The amount of *PARK2* transcript in Pt1 was similar to controls, whereas in the other mutant fibroblasts transcript levels are decreased (~40% in Pt2, ~25% in Pt3%, ~50% in Pt4). **(C)** Western blot analysis using whole-cell lysates from control and mutant fibroblasts. Protein levels of Parkin were estimated using an antibody able to recognize the C-terminus of protein. Vinculin was used as loading control. **(D)** Densitometric analysis of Parkin levels normalized to Vinculin, obtained from three independent western blot experiments. The controls' mean is set equal to 100%. Strongly reduced levels of Parkin were detected in mutant fibroblasts; ~20% in Pt1, ~10% in Pt2 and Pt3, <5% in Pt4.

To examine the possible effect of *PARK2* mutations on splicing, a PCR amplification was performed on mRNA extracted from patients' fibroblasts. The full-length transcript was observed for Pt1, Pt2, Pt3 mutant fibroblasts, whereas a shorter transcript, corresponding to a *PARK2* isoform lacking exon 3-4-5 (sequence data not shown), was observed in Pt4 (Figure 1A). Subsequently a quantitative PCR was performed. Pt2, Pt3, and Pt4 showed a significantly decrease of *PARK2* transcript less than 50%, while Pt1, carrying two missense mutations, presented an amount of transcript similar to controls (Figure 1B). However, by Western blotting analysis Parkin protein was significantly reduced in all samples including Pt1; this suggested instability of Parkin species with missense changes (Figures 1C,D).

### Oxygen consumption is increased in *PARK2*-mutant fibroblasts and not correlated to ATP cellular levels

**Figure 2:**
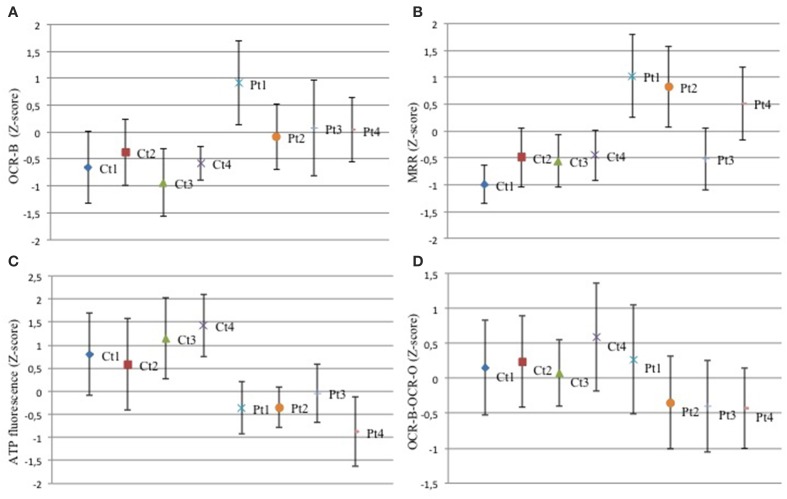
**Mitochondrial function. (A–D)** The z-score values (dots) for basal oxygen consumption (OCR-B), maximal respiration rate (MRR), ATP amount, OCR-B minus OCR-O, are shown for control and mutant fibroblasts. Three independent experiments, each with 16 replicates, were performed. One-Way ANOVA followed by Tukey test was used (controls vs. mutant). **(A)** Mutant fibroblasts exhibited a significant increment of oxygen consumption. Pt1: *p* < 0.01 vs. Ct1,Ct2,Ct3,Ct4. Pt2: *p* < 0.05 vs. Ct1; *p* < 0.01 vs. Ct3. Pt3: *p* < 0.05 vs. Ct1; *p* < 0.01 vs. Ct3, Ct4. Pt4: *p* < 0.05 vs. Ct1, Ct4; *p* < 0.01 vs. Ct3. **(B)** With the exception of Pt3, mutant cells showed a higher MRR. Pt1, Pt2, Pt4: *p* < 0.01 vs. each Ct. **(C)** Analysis of ATP cellular levels showed a significant reduction in all mutant fibroblasts respect to controls. Pt1, Pt2, Pt4: *p* < 0.01 vs. each Ct; Pt3: *p* < 0.05 vs. Ct1, Ct2; *p* < 0.01 vs. Ct3, Ct4. **(D)** The difference between OCR-B and OCR-O has a trend toward reduction in mutant fibroblasts respect to controls. Pt1: *p* < 0.05 vs. Ct4; Pt2, Pt3, Pt4: *p* < 0.05 vs. Ct1, Ct2, Ct4.

In order to investigate mitochondrial bioenergetics status, we evaluated respiration and extracellular acidification by microscale oxyghraphy. We consistently observed a significant increment of oxygen consumption (basal OCR-B) in all mutant samples respect to controls (Figure 2A). Also the ratio OCR/ECAR was higher in mutant respect to controls whereas ECAR data was comparable between controls and mutant cells (Supplementary Figures S2A,B), indicating that metabolism of mutant cells did not shift toward glycolysis and that oxygen consumption alteration was directly linked to electron transfer chain activity. Similarly, MRR which is an index of electronic chain transport efficiency, was higher than controls in all mutant fibroblasts, except for Pt3 (Figure 2B). These findings indicated that mutant cells forced the activity of the respiratory chain in order to produce energy.

Then, we investigated the ATP content. Despite the increased respiratory parameters, we observed a significant reduction of ATP cellular levels in all mutant fibroblasts suggesting that oxygen consumption was uncoupled to ATP cellular biosynthesis (Figure 2C). We verify the dependence of oxygen consumption on ATP synthase activity, evaluating the difference between OCR in basal condition (OCR-B) and after complex V block (OCR-O). In mutant samples, except for Pt1, these values were lower than controls (Figure 2D). This result reflects reduced sensitivity to oligomycin (Supplementary Figure S2C) and suggests that mutant fibroblasts had uncoupled mitochondria.

### Mutant fibroblasts showed altered mitochondrial membrane potential not associated with mitochondrial fragmentation

**Figure 3:**
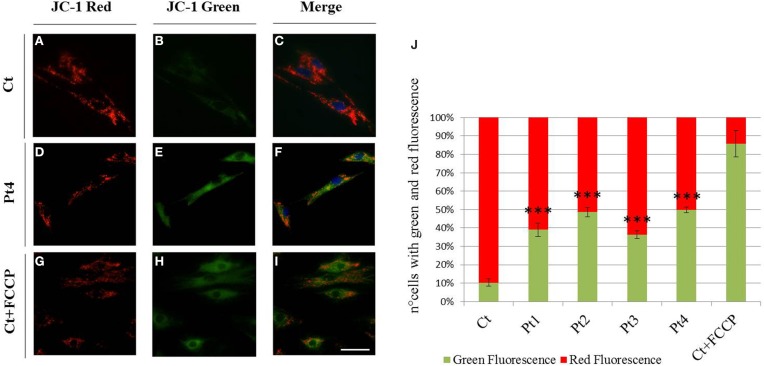
**Mitochondrial membrane potential (ΔΨm). (A–I)** JC-1 staining on fibroblasts from controls **(A–C)**, Pt4 **(D–F)** and controls treated with the uncoupling agent FCCP (1 μM, 10′) **(G–I)**. Red fluorescence **(C)**, sign of preserved ΔΨm, was observed in almost all non-treated controls, whereas several mutant fibroblasts **(E,F)** as well as FCCP treated controls, displayed green fluorescent signals, index of mitochondrial membrane depolarization **(H,I)**. Scale bar, 50 μm. **(J)** Quantification of cells observed with green and red fluorescence (expressed in percentage). Data were from three independent experiments, each with 50 counted cells. Mutant fibroblasts (Pt1, Pt2, Pt3, Pt4) presented a higher number of cells with green fluorescence (~40–50%), than controls (~10%). Test Chi^2^-Pearson, Pt vs Ct; p: 2,49E-06 (Pt1); p: 2,83E-09 (Pt2); p: 1,37E-05 (Pt3); p: 1,21E-09 (Pt4). ^***^ *p* < 0.0001.

To evaluate the mitochondrial membrane potential we used JC-1 staining. Cells with high mitochondrial membrane potential promote the formation of red fluorescent JC-1 aggregates, while cells with low membrane potential show a diffuse green fluorescence. As shown in Figure 3 the vast majority of the fibroblasts derived from controls showed a clear red fluorescence (Figures 3A–C), while we observed a diffuse green fluorescence (Figures 3D–F) in a great percentage of mutant *PARK2* fibroblasts (Figure 3J) indicating the presence of altered mitochondrial membrane potential. To validate the assay, we treated controls fibroblasts with uncoupling agent FCCP to induce ΔΨm loss; as expected, treated fibroblasts displayed green fluorescent signals, whereas untreated fibroblasts showed red fluorescent aggregates (Figures 3G–I and in Figure 3J).

**Figure 4:**
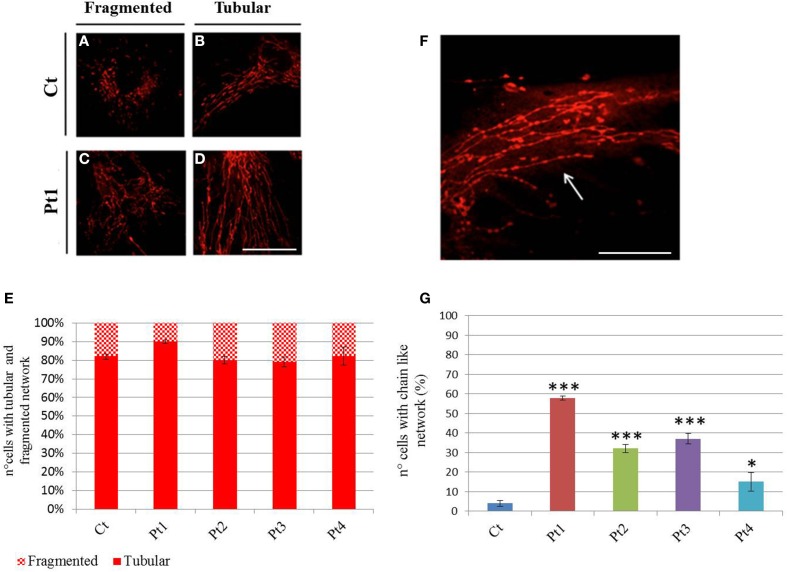
**Mitochondrial network. (A–D)** Mitotracker Red staining from controls **(A, B)** and mutant Pt1 **(C, D)**. Representative images of cells presenting fragmented or tubular network are shown. Scale bar, 50 μm. **(E)** Graphic representation of cells from control and patients, with fragmented or tubular network. The network morphology was assessed by Metamorph analysis: cells with shape factor ≥0.650 were classified as fragmented network, cells with shape factor <0.650 as tubular network. Data were from three independent experiments, each with 50 counted cells. No difference on mitochondrial network was observed between mutant fibroblasts (Pt1, Pt2, Pt3, Pt4) and controls (Ct). Test Chi^2^-Pearson (Pt vs Ct) was performed and resulted not-significant for all mutant fibroblasts; p: 0,10 (Pt1); p: 0,72 (Pt2); p: 0,59 (Pt3); p: 0,97 (Pt4). **(F)** Mitotracker Red staining showing a peculiar mitochondrial “chain-like” network (arrow), observed virtually only in mutant fibroblasts. Scale bar, 100 μm. **(G)** Quantification of cells with “chain-like” structure after Mitotracker Red staining. Data were from three independent experiments, each with 50 counted cells. Test Chi^2^-Pearson (Pt vs. Ct); p: 1,83E-16 (Pt1); p: 2,56E-07 (Pt2); p: 7,46E-09 (Pt3); p: 0,008 (Pt4). ^***^ *p* < 0.0001; ^*^ *p* < 0.01.

We next visualized the mitochondrial networks by Mitotracker Red, a mitochondrion specific dye. First we determined the form factor of cells by MetaMorph analysis, classifying the cells into two groups: fragmented network (Figures 4A–C), or tubular network (Figures 4B–D). This morphological assessment showed no difference between mutant and control individuals under basal condition (Figure 4E). However, we observed a peculiar network with “chain-like” structure (Figures 4F,G) virtually only in mutant fibroblasts.

## Discussion

Several reports suggesting a connection between (dys)function of Parkin and mitochondria quality control have been published. However, the use of cellular models based on overexpression of Parkin or its partners, and the presence of recombinant tagged proteins, could have introduced bias or artifacts in various studies (Burchell et al., 2012; Rakovic et al., 2013). To better define the function of Parkin we use a physiological model, investigating mitochondrial dysfunction in fibroblasts derived from four patients characterized by JPD and carrying recessive *PARK2* mutations. Till now the studies performed on *PARK2* mutant fibroblasts in order to explore the impact of Parkin on mitochondrial functionality, have remained vague. Despite the small group of samples, we were able to analyse how heterogeneous mutations likely affect mitochondrial homeostasis. We demonstrated that *PARK2* mutations present in our group of patients drastically reduced the Parkin protein levels, thereby giving us Parkin knock-down or knock-out cellular models.

**Figure 5:**
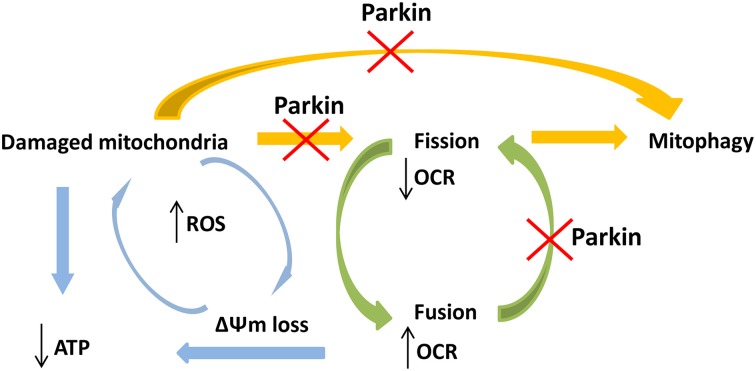
**Schematic view of the hypothetical connections between *PARK2* mutations and impairment of mitochondrial functions**. *PARK2* mutations impair Parkin functionality and linked pathways, like mitophagy and fission process. Dysfunction in Parkin may interfere in both these pathways implying an accumulation of damaged mitochondria, with ATP loss. Mutations in *PARK2* may also compromise the physiological mitochondrial fission process. This implies that mitochondria are forced to stay in a fuse state, with high respiration rate and impaired membrane potential (ΔΨm), causing ROS increase and subsequently damage to mitochondria. These events create a vicious circle where not only the physiological damaged mitochondria were not removed but, in absence of Parkin, also newly synthesized mitochondria are more likely prone to accumulate damages.

Previous studies reported mitochondrial respiratory chain (MRC) complex I deficiency in leukocytes derived from patients with *PARK2* mutations (Müftüoglu et al., 2004), but similar analyses on fibroblasts have resulted in contradictory outcomes (Mortiboys et al., 2008; Grünewald et al., 2010). We tried to use a more sensitive assay in a physiological condition, investigating cellular respiration on intact vital cells, since its rate depends on the cumulative activities of the entire set of MRC complexes (Steenweg et al., 2012). In Parkin-mutant fibroblasts MRR, which indicated efficiency of electron transport chain, resulted augmented or similar respect to controls suggesting absence of defects in mitochondrial complexes, in agreement with previous work of Grünewald and colleagues. We observed also a high basal oxygen consumption, indicating elevated electron flow through the respiratory chain with an expected increase of oxidative phosphorylation. The absence of acidification indicated there was not a shift toward glycolytic metabolism. However, surprisingly, we observed significantly reduced ATP cellular concentration in Parkin-mutant fibroblasts (Grünewald et al., 2010; van der Merwe et al., 2014) and, also, an independence of oxygen consumption from the activity of complex V (ATP synthase). This discrepancy suggested a loss of mitochondrial membrane potential, reflecting the loss of coupling between oxygen consumption and ATP synthesis. Accordingly, in Parkin-mutant fibroblasts even under basal conditions, we found an impairment of ΔΨm, a feature previously reported (Mortiboys et al., 2008; Grünewald et al., 2010). The presence of uncoupled mitochondria impairs ATP production, essential for cellular vitality; in addition, the uncoupling can lead to elevated production of reactive oxygen species (ROS), due to compromised mitochondrial antioxidant mechanisms (Aon et al., 2010), with increased levels of oxidized proteins and eventually cell death (Grünewald et al., 2010; Pacelli et al., 2011). In order to prevent this oxidative damage, cells activate pathways able to selectively eliminate damaged mitochondria (Lemasters, 2005; Frank et al., 2012). Several studies have showed that Parkin plays a pivotal role in mitophagy through a defined mechanism triggered by loss of ΔΨm. To ensure that only faulty mitochondria are removed, a mitochondrial fission process precedes mitophagy, permitting the isolation of damaged organelles from remaining healthy network (Twig et al., 2008; Chan et al., 2011; Wang et al., 2011). Different studies on human cells also demonstrated an impact of Parkin mutations on mitochondrial network and fission/fusion dynamics but the results are still debated. Parkin mutant cells were found to be comparable to controls (Grünewald et al., 2010; van der Merwe et al., 2014) or more prone to enter fusion with increase in mitochondrial branching (Mortiboys et al., 2008); only in one study fragmented mitochondrial network was observed (Pacelli et al., 2011). In agreement with previous works (Grünewald et al., 2010; van der Merwe et al., 2014) we detected no significant difference in mitochondrial network between Parkin-mutant and control fibroblasts in spite of the presence of altered mitochondrial potential. Our results suggest that in absence of Parkin mitochondrial fission is partly impaired implying that damaged mitochondria might injury the remaining intact network. According to several publications ascribing Parkin a role in promotion of mitochondrial removal after mitochondrial depolarization, an impairment of Parkin functionality could affect Parkin/PINK1 dependent mitophagy, thus implying the accumulation of damaged mitochondria (Figure 5). Interestingly, we observed a “chain like” network only in Parkin mutant fibroblasts. This data could indicate that, because of altered mitochondrial membrane potential, the fission mechanism was initialized but, in absence of Parkin, was not concluded, suggesting that Parkin plays a role in a late-stage of fission process. In agreement with our results, Parkin has been recently shown to act on mitochondrial fission protein Drp1, promoting Drp1-dependent mitochondrial fission by a mechanism that seems to be independent from PINK1 (Buhlman et al., 2014). Considering our results and the above mentioned article (Buhlman et al., 2014), we hypothesize that mutations in Parkin, and hence an impairment of its activity, impact not only directly on mitophagy but can also affect the physiological regulation of mitochondrial dynamics. Recent evidences suggest that mitochondrial dynamics can regulate mitochondrial activity and augmented fusion of mitochondrial network increase respiratory activity (MacVicar and Lane, 2014; Mishra et al., 2014). We speculate that, in absence of Parkin, mitochondria are forced to stay in a “fuse” state, with high respiration rate but uncoupled to ATP production and associated with altered ΔΨm; this may produce increase of ROS with subsequently damage to mitochondria (Figure 5). The limited number of analyzed patients requires confirmatory studies.

However, our study confirms that *PARK2* mutations lead to mitochondrial impairment, in particular reduction in ATP cellular levels and alteration of ΔΨm, even in non-neuronal cells in agreement with previous studies (Mortiboys et al., 2008; Grünewald et al., 2010; van der Merwe et al., 2014). Further investigation of the relationship between Parkin and mitochondrial dynamics/mitophagy is warranted. Indeed, this study provides that skin fibroblasts from *PARK2* mutant patients may be a suitable system to study mitochondrial dysfunction in PD and to test new therapeutic approaches based on rescue of mitochondrial phenotypes.

### Conflict of interest statement

The Guest Associate Editor Tiziana Lodi declares that, despite having collaborated with the author Daniele Ghezzi, the review process was handled objectively. The authors declare that the research was conducted in the absence of any commercial or financial relationships that could be construed as a potential conflict of interest.
