# Supplementary material for: Mitochondrial dysfunction in Parkinson disease: evidence in mutant PARK2 fibroblasts
Source: Front Genet. 2015 Mar 11;6:78. doi: 10.3389/fgene.2015.00078 (PMC4356157; doi:10.3389/fgene.2015.00078)
Supplement: Supplementary file 1 [file Image1.PDF]

Supplementary Figure 1

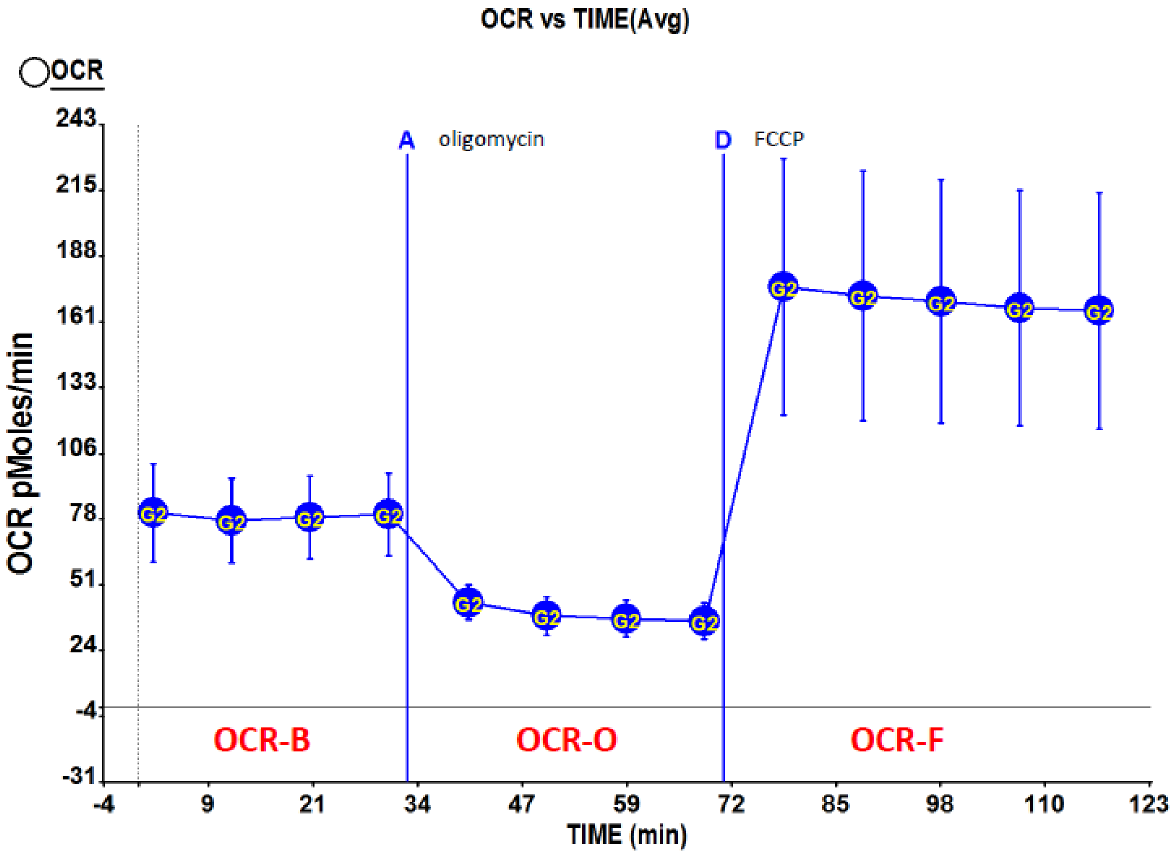

Figure S1. Representative trace of oxygen consumption rate during total experiment.

Oxygen consumption rate (OCR) was measured in basal conditions (OCR-B); after injection of oligomycin, an inhibitor of complex V (OCR-O); and after injection of FCCP, a ΔΨ dissipator that permits maximal mitochondrial respiration in uncoupled mitochondria (OCR-F). Four measurements were performed for each condition, using 12-16 replicates.

Supplementary Figure 2

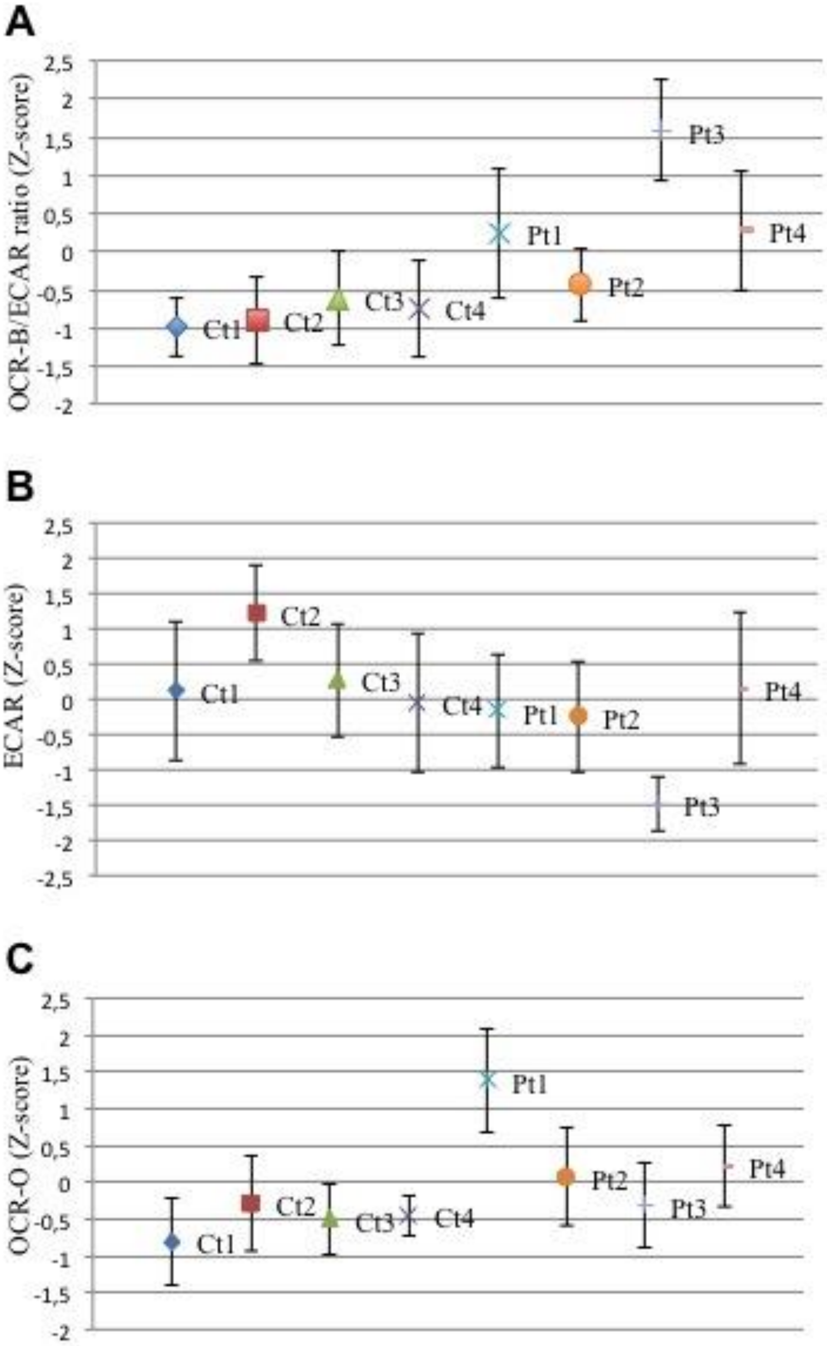

Figure S2 Measurement of extracellular acidification rate and sensitivity to oligomycin

(A)The ratio between oxygen consumption rate (OCR) and extra cellular acidification rate(ECAR) was higher in mutant respect to control cells. Pt1, Pt3, Pt4: $p<0.01$ vs each Ct; Pt2: $p<0.05$ vs Ct1. One-way Anova followed by Tukey test was used

(B) ECAR data was variable between mutant and controls; only Pt3 was consistently lower than controls ($p<0.01$ vs each control). Pt1 and Pt2 were significantly different only from Ct2 ($p< 0.05$), while for Pt4 no difference vs controls was found.

(C) Oxygen consumption rate after oligomycin injection (OCR-O) was higher in all mutant cells but Pt3. Pt1: $p<0.01$ vs each Ct. Pt2: $p< 0.01$ vs Ct1, Ct3; $p< 0.05$ vs Ct4. Pt4: $p< 0.01$ vs Ct1; $p< 0.05$ vs Ct3, Ct4.
